# Nature and prevalence of adverse drug reaction of antiretroviral medications in Halibet National Referral Hospital: a retrospective study

**DOI:** 10.1186/s40360-019-0307-9

**Published:** 2019-05-06

**Authors:** Lidya Hagos, Siyoma Fessehaye, Indermeet Singh Anand

**Affiliations:** Asmara College of Health Sciences, P.O box – 1833, Asmara, Eritrea

**Keywords:** Anti-retroviral medications, Adverse drug reaction, Nature, Prevalence, Seriousness, Risk factors

## Abstract

**Background:**

Monitoring the safety of antiretroviral therapy (ART) remains a challenge in resource-constrained countries such as Eritrea due to their serious adverse drug reactions (ADRs). This study was aimed at assessing the prevalence, nature, seriousness and possible risk factors of ART associated ADRs in Halibet National Referral Hospital in Eritrea.

**Method:**

A three month retrospective, longitudinal, descriptive study of patients treated with ART between September 2005 and December 2016 was conducted in Halibet National Referral Hospital. Demographic characteristics, treatment details, reaction and outcome details, laboratory investigations and other information was abstracted manually from patients’ clinical cards. Statistical analysis was conducted using both univariate and multivariate analysis and statistical significance was tested using 95% confidence intervals and/or *p*-value. A *P*-value < 0.05 was regarded as being statistically significant.

**Results:**

Of the 309 patients screened, 62.8% encountered at least one ADR and 29.8% of the reactions were serious with similar male to female ratio. Gastrointestinal symptoms were the most common ADR and were associated mostly with Atripla followed by AZT + 3TC + NVP drug combinations, but lipodystrophy followed by peripheral neuropathy which were both commonly associated with Stavudine and anemia associated with Zidovudine were the most serious. Patients with CD4 count below 200 were more likely to develop ADRs (*p* = 0.000).

**Conclusion:**

ADRs associated with ART drugs in Halibet hospital were found to be highly prevalent. Furthermore, CD4 count below 200, was identified as a major risk factor that predisposes patients to ADRs. This is burdensome to resource constrained countries such as Eritrea who have limited drug options and high HIV prevalence, therefore these findings will help patients and healthcare professionals understand the nature as well as seriousness of these ADRs and identify the risks involved with ART medications which can help minimize ART associated ADRs early on.

## Background

Highly Active Anti-Retroviral Therapy (HAART) has made a significant change in the lives of people living with HIV (PLWH) in decreasing AIDS-related deaths and improving quality of life [1]. Despite their remarkable contribution, these drugs have been associated with serious adverse drug reactions (ADRs) that may lead to drug resistance and switching of anti-retroviral therapy (ART) regimen [2, 3] and emergence of new comorbidities which may lead to decreased adherence consequently leading to virological failure [4, 5]. It has been elucidated that the type of ART regimen influences the timing, nature and duration of ADRs [6, 7]. Furthermore, the occurrence of ADR might be higher in developing countries due to higher prevalence of concomitant conditions, overstretched healthcare systems and economic constraints that would hamper close follow up of patients on HAART [8–10]. Moreover, age, gender and the disease itself have been identified as risk factors for ADRs of HAART in different countries. [6, 11].

Limited study has been conducted on the adverse effect of ART in Sub-Saharan countries, such as Eritrea, despite having high prevalence of HIV. A five-month prospective study conducted in Eritrea by Russom et al. 2017 [12] aimed at measuring ADR related hospital admissions in all Eritrean hospitals showed first line ART drugs (Zidovudine/lamivudine and Tenofovir/lamivudine) to be among the top three drugs implicated in causing ADR related deaths. The study further added that ART associated anemia accounted for 20.8% of the ADR related deaths which could have easily been prevented by appropriate laboratory monitoring. Despite the above facts, no study has been conducted so far in Eritrea to evaluate the safety of ART. The aim of this study was, therefore, to determine the prevalence, nature, seriousness and risk factors of adverse reactions of antiretroviral drugs.

## Methods

### Study design and setting

This was a retrospective, historically longitudinal, descriptive study conducted in all eligible patients treated with ART in Halibet national referral hospital. Longitudinal data (drug and medical history) of patients compiled between September 2005 and December 2016 in the ART clinic was abstracted from patients’ clinical cards. The HIV clinic in Halibet hospital as part of the infectious clinic gives an outpatient services to 1242 adult patients, above 18 years of age, that come from different parts of the country.

## Source and study population

The study population was PLWH/AIDS who were attending Halibet national referral hospital’s ART clinic between 2005 and December 2016. The source population covers all HIV patients in the country as the hospital provides services to all patients coming from different parts of the country. Patient cards that were hard to assess were excluded from this study since the information they presented was not enough to be evaluated. Furthermore, patients aged 60 years and above were also excluded from the study as they may have different co-morbidities and are likely to take multiple drugs which would make the causality assessment more challenging.

### Exposure definition

Once a patient is diagnosed with HIV, he/she may start on HAART. HAART refers to the combination of three or more antiretroviral drugs for the treatment of HIV infection. The patients may start therapy regardless of their CD4 count under the new ART guidelines. Once patients decide to start ART, they begin with standard first-line HAART regimen which for an adult includes Atripla (Tenofovir /Emtricitabine/Efavirenz), Tenofovir/Lamivudine+Nevirapine, Zidovudine/Lamivudine+Nevirapine, Zidovudine+Lamivudine+Efavirenz, Abacavir+Lamivudine+Efavirenz, Abacavir+Lamivudine+Nevirapine. Patients who develop resistance to these first-line regimens(clinical failure, immunological failure and virological failure) move to a boosted protein inhibitor (PI) and two nucleoside reverse transcriptase inhibitor (NRTI) combinations as they are preferred strategy for second-line ART. In case of TDF +3TC or FTC based first-line regimen failure, AZT + 3TC + LPV/r or AZT + 3TC + ATV/r are used. For AZT + 3TC based first-line regimen failure, TDF + 3TC/FTC + ATV/r and TDF + 3TC/FTC + LPV/r are used as second-line regimens respectively. Since these are combination drugs, they do not use weight-based regimen and the same doses are given to all patients deemed as adults. In case of ADRs, specific or symptomatic relief may be given to alleviate the encountered ADRs and in severe cases the drug may be withdrawn.

### Outcome definition

This study’s primary outcome measures were the nature of ADRs encountered, their prevalence, seriousness as well as the possible risk factors that predispose patients to ADRs.

### Sampling, study instruments and data collection approach

A sample that would ideally be representative of the patients in Halibet National referral hospital was taken. This sample was calculated using the following formula:

$$ Initial\ sample\ size=\frac{Z^2 pq}{e^2}=384.16 $$

Adjusted to the population size (population correction factor) = $\mathrm{nl}\frac{N}{N+n1}=293$.

Where p = prevalence of ADR = 0.05.

q = complement of *p* = 1–0.05.

Z = parameter of the confidence interval = 1.96.

e = margin of error = 0.05.

N = population size = 1242.

Three hundred nine patients were taken as sample to further decrease the margin of error.

This sample was taken from the population by selecting every fifth card from the 1242 cards that were alphabetically ordered to ensure randomness. Data collection tools developed by the Eritrean Pharmacovigilance Centre for conducting similar studies were used to capture data. Demographic information of all the eligible patients attending the ART clinic during the study period was documented using a ‘Patient Listing Form’. It was used to capture demographic information of the patients regardless of their adverse drug reaction status including age, sex, number of drugs taken, availability of co-morbidities, CD4 count and so on. The second data collection tool was a comprehensive and well-structured tool for those with ADRs aimed at collecting patient demographic information, reaction details and drug details on both suspected and concomitant drugs including those taken for longer period of time to manage ADRs. Furthermore, detailed information of ADRs manifested during treatment including type of ADR, date reaction started, date reaction stopped, therapy started and stopped, dechallenge and rechallenge information, seriousness of the reactions, reaction outcome, information on other possible alternative cause(s) or explanations, management taken and treatment provided whenever available was recorded.

For the purpose of this study, the definition of an ADR was based on the one developed by the World Health Organization (WHO) [13] WHO has defined an ADR as a noxious, unintended drug reaction that occurs at doses normally used in humans for prophylaxis, diagnosis or therapy. Depending on the definition used, therapeutic failure, overdose or poisoning and drug abuse was excluded from the study.

## Case assessment

Causality assessment was performed for all suspected ADRs using Naranjo probability scale [14]. The Naranjo ADR probability scale is a tool developed to assess the probable causal associations between the suspected drug(s) and the ADRs encountered. It consists of a series of 10 questions which are believed to be among the main features of causality including whether the event is documented, plausible temporal association, dechallenge and rechallenge information, likelihood of alternative causes, dose-response relationship, presence of objective evidence, history of similar problems before with the same or similar medications and so on. The questions are answered as either “Yes”, “No”, or “Do not know”. Different values are allocated for each question according to its importance as − 1, 0, + 1 or + 2. Based on the total score, assessors categorize the likelihood of drug-reaction relationship as “Certain”, “Probable”, “Possible” or “doubtful/unlikely”.

Seriousness of the identified suspected ADRs was determined according to the definition of the ICH E2A guideline [15]. According to the ICH E2A guideline, a serious adverse event or reaction is any untoward medical occurrence that at any dose:

- Resulted in death,
- Is life-threatening,
- Required hospitalization or resulted in prolongation of existing hospitalization,
- Resulted in persistent or significant disability/incapacity,
- Caused congenital anomaly/birth defect or
- Medically important event or reaction that required medical/surgical intervention to prevent serious outcome.

### Data processing and statistical analysis

Data was captured into computer using an entry program developed with CSPro version 6.3.2 software package. All questionnaires were entered twice; that is 100% verification was done to eliminate keying errors during entry. Data was edited during and after data entry using CSPro and Statistical Package for Social Science version 20 (SPSS-20). Both descriptive and analytical analysis was carried out on the data using SPSS. Both univariate and multivariate analysis were carried out to measure the association of some potential risk factors and ADRs. Results were presented as percentage and frequencies as appropriate. To test statistical significance, 95% confidence intervals and/or *p*-value were used. A *P*-value < 0.05 was regarded as being statistically significant.

## Results

Three hundred and nine (309) patients, 64.1% females and 35.9% males, were included in this study (Table 1).

**Table 1:** Background characteristics of all patients included in study

Patient background characteristics		Number	%
Sex of the patient	Male	111	35.9
	Female	198	64.1
Broad age group	< 25	16	5.2
	25–34	93	30.1
	35–44	135	43.7
	45 and above	65	21.0
Any co-morbidities	Yes	70	22.7
	No	239	77.3
CD4 count	Below 200	197	63.8
	200–349	81	26.2
	350 and above	28	9.1
	Not Reported	3	1.0
Weight of patients (Kg)	Below 50	91	46.9
	50–59	66	34
	Above 60	37	19.1
	Total	309	100.0

**Fig. 1:**
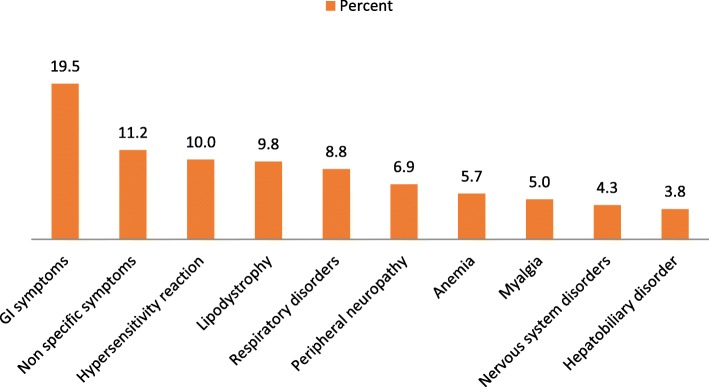
The frequency and nature of all ADRs that were seen in descending order

**Fig. 2:**
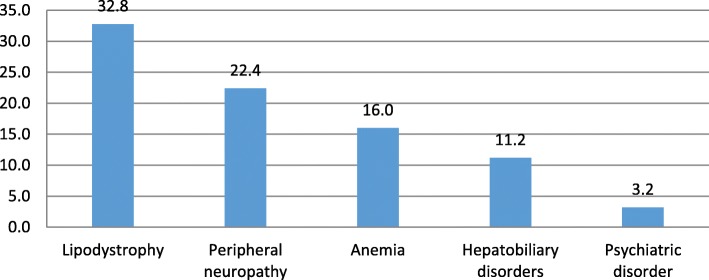
Frequency of serious ADRs

Of the 309 patients, 62.8% experienced at least one ADR. 128 (64.6%) of these patients were females while 66 (59.5%) were males. 44.3% of them experienced at least three ADRs with similar male to female ratio. Of these, 29.8% were found to be serious. Gastrointestinal upset (19.5%) were the most frequently reported ADR followed by non-specific symptoms (11.2%), hypersensitivity reactions (10%) and lipodystrophy (9.8%) (Fig. 1). Lipodystrophy (32.8%) followed by peripheral neuropathy (22.4%) and anemia (16%) were among the frequently reported serious reactions (Fig. 2).

Patients with CD4 count, below 200 cells/mm3, and between 200 and 348 had a statistically higher occurrence of ADRs, 65.5 and 67.9%, respectively, (× 2 = 18,539, *P* = 0.000). Those with CD4 count below 200 were about six times more likely to face ADRs compared to those with higher CD4 count (OR = 6.2; *p* = 0.000), and those patients with CD4 count between 200 and 349 were also about five times more likely to develop ADRs (OR = 5.1; *p* = 0.001) compared to patients with CD4 count above 349. In the regression model patients with age (35–44 years old), female gender, lower baseline body weight, and co-morbidities were tested to have statistically insignificant impact (Table 2). The regression model fitted to the data was statistically significant in predicting the likelihood of ADR among the patients (*p* = 0.002 < 0.05). Therefore the model correctly predicts the ADR status of the patients (69.6%).

**Table 2:** Prevalence of ADR based on background characteristics

Background characteristics		Prevalence of ADR		Significance test (*P*-value)
		Number	%	
Broad age group	< 25	9	56.3	
	25–34	51	54.8	
	35–44	93	68.9	0.175
	45 and above	41	63.1	
Sex of the patient	Male	66	59.5	
	Female	128	64.6	0.365
Any co-morbidities	Yes	49	70.0	0.156
	No	145	60.7	
CD4 count	Below 200	129	65.5	
	200–349	55	67.9	0.000
	350 and above	7	25.0	
	Not Reported	3	100.0	
	Total	194	62.8	

In terms of time of onset of the reactions, anemia had an earlier onset with 52.2% of the reaction starting earlier than a year. Similarly, 39.55% of hypersensitivity reaction, 66.7% of hepatobiliary disorder, and 53.6% peripheral neuropathy had higher occurrence within the first year of treatment. Lipodystrophy, however, didn’t start to appear until two to three years of initiating the ART regimen (41.5%), while the rest occurred after 3 years (58.5%).

Most of the reactions were associated with Stavudine and Zidoudine containing HAART with 75.2 and 67.5% respectively. Tenofovir and Nevirapine were similar in bringing ADRs with 54.1 and 53.5% respectively. Lamivudine is the least offending drug with 46.8%.

**Fig. 3:**
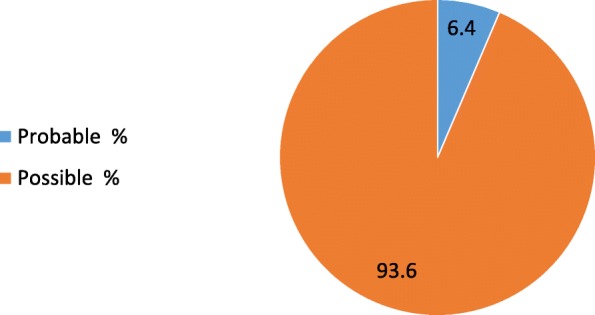
The drug-reaction relationship determined using Naranjo-probability scale

The causal relationship that was assessed using Naranjo probability scale in majority of the suspected adverse drug reactions cases was found to be possible 393/420. (Fig. 3).

The suspected offending drug was withdrawn in 21.1% of the cases to manage the encountered ADRs. In the rest of the cases, either management was not documented or no action was taken. Specific treatment was given in 24% of the ADRs 29% were managed symptomatically, and 2.9% were managed both with specific treatment and symptomatically.

## Discussion

This study found that ADR associated with ART medications are highly prevalent. This is similar with previous studies [16, 17].

GI symptoms were the most commonly reported ADRs similar to previous studies [16, 18, 19] followed by non-specific symptoms such as chills, fever, headache, hypersensitivity reactions and lipodystrophy. Furthermore, lipodystrophy which was associated primarily with Stavudine was the most serious ADR followed by peripheral neuropathy, anemia and hepatobiliary disorders. However, it is important to remember that Stavudine has been removed from ART list of medications and thus lipodystrophy is not as prevalent as it once was. Peripheral neuropathy was associated with Stavudine followed by Zidovudine and in one case by Atripla, this is an important finding because Atripla hasn’t been known to cause peripheral neuropathy and only one other study to our knowledge has similar findings [20] (Table 3).

**Table 3:** ADRs that were associated with specific drug combinations are shown below

Nature	Drug	%
GI upset	Atripla	32.9
	AZT + 3TC + NVP	32.9
Peripheral neuropathy	D4T	65.5
	AZT	27.6
	Atripla	6.89
Anemia	AZT	70.8
	Atripla	16.6
	D4T + 3TC + NVP	8.3
Hypersensitivity reaction	NVP	61.9
	EFV	23.8
Hepatobiliary disorder	NVP	62.5
	EFV	31.25
Lipodystrophy	D4T	100%

Anemia which was mostly associated with Zidovudine was the third most serious ADR, unlike previous studies that found anemia to be the most serious ADR [12, 16, 21]. This difference could be either due to the inappropriate laboratory monitoring of the patients on HAART in Halibet hospital which undermines the real frequency or due to the nature of this study. Hepatobiliary disorder was associated with Nevirapine and to a lesser extent with Efavirenz. This was consistent with a systematic review and meta-analysis conducted by Shubber et al. [22]. Most of the ADRs (anemia, hypersensitivity reaction, hepatobiliary disorder and peripheral neuropathy) were observed within the first year of starting ART while lipodystrophy occurred within 2–3 years following commencement of the ART. The difference in the timing of these ADRs depends on the type of drug regimen [6, 23, 24] nature and pathophysiology of the reactions [18].

Females had been previously recognized to have a statistically significant ADR occurrence associated with ART medications [18, 19, 25] which differed from this study’s findings.

Low CD4 count was identified to be a risk factor with the occurrence of ART related ADR. The finding of this as a risk factor has been controversial across studies where some studies were consistent with our findings [26] while others reported that patients with higher baseline CD4 count were more likely to develop ADRs compared to those with lower CD4 count [16, 18, 25]. Some studies believe that the prevalence of these ADRs depend on the type of medications. For instance, Nevirapine associated hepatotoxicity increases in patients with higher CD4 count [5, 27] while Zidovudine associated anemia [5] and Stavudine associated lipodystrophy is higher in patients with lower CD4 count [28, 29]. However, a study done by Shelburne et al. [30] indicates that the reason ADR is seen in low CD4 count patients is because of a rapid rise of CD4 lymphocytes soon after ART initiation which can be an important stimulus of ADR as part of an immune reconstitution inflammatory syndrome (IRIS).

When attempting to establish a causal relationship, majority of the cases were of a possible association. This may be due to the methodological nature of the study, being a retrospective one, moreover, it may be due to the fact that HIV disease itself is associated with some of the ADRs that are associated with ART medications.

One of the inherent limitations of this study was that it was a retrospective study. Moreover, we only included patients aged 60 years and below which might introduce selection bias into the study. Additionally, medical cards that were difficult to assess were not included. Several factors that may predispose the patients to ADRs similar to antiretroviral medications including non-antiretroviral co-administered medications such as over the counter drugs and patients life style (alcoholism or smoking) were not documented in the patients’ clinical cards and thus their contribution to ADR prevalence was left unmeasured. Furthermore, co-morbidity types were not captured by our questionnaire as it lacked to address this. In addition, the findings are from a small size and from one referral hospital, hence a bigger study needs to be conducted to solidify these findings.

## Conclusion

The findings in this study have proven that ART associated ADRs are highly prevalent and are threatening the adherence level that is much needed to ensure maximum benefit from these medications. The drugs as well as the risk factors that are mostly associated with ADR occurrence found in this study should help health professionals at all levels to foresee, identify and minimize ADR at the earliest possible time as well as to understand the need for close follow up and monitoring to avoid the occurrence of serious ADRs.

## Abbreviations

3TC: Lamivudine
ABC: Abacavir
ADR: Adverse Drug Reactions
ART: Anti Retroviral Therapy
ATRIPLA: TDF + 3TC + EFV
AZT: Zidovudine
D4T: Stavudine
EFV: Efavirenze
FTC: Emitricitabine
HAART: Highly Active Antiretroviral Therapy
HIV: Human Immune Deficiency
ICH: International Conference on Harmonization
IRIS: Immune Reconstitution Inflammatory Syndrome
LPV/r: Lopinavir/ Ritonavir
NRTI: Nucleoside Reverse Transcriptase Inhibitors
NVP: Nevirapine
PI: Protease Inhibitor
PLWH: People Living With HIV
TDF: Tenofovir
